# Bacillus Tuberculosis*Buffalo Microscopical Club, Dec. 12th, 1882.

**Published:** 1883-03

**Authors:** Mary B. Moody


### Bacillus Tuberculosis.*

Mary B. Moody, M. D.

That a malady so widely disseminated and so unmanageable as the tubercular variety of phthisis pulmonalis, or what is popularly known as consumption, must, in its beginnings, be microscopic in character, has for years been suspected by students of medicine. That the patient and persistent labors of one individual, Dr. Robert Koch, should at last have revealed it, may be almost, but is not quite, beyond belief. The fullness of time for such a discovery had been indicated by the great amount of research, in regard to both the pathology and the etiology of the disease. The revelations of Davaine's and Pasteur's studies of anthrax and of Klebs' and Tommassi-Crudelli's of malarial disorders, were brilliant and suggestive. Doubtless, the discovery and investigation of bacillus anthracis and of bacillus malaria, led to the discovery of bacillus tuberculosis.

Botanically, the bacilli belong to the lowest known orders of the vegetable kingdom. Their possession of cellulose determines this point. Many of the earlier observers of bacteria, were, for a long time, in doubt with regard to their classification. They were believed to be the lowest of animal organizations. Their classification in the vegetable kingdom has been decided by the absence of chlorophyll. They are not algae but fungi, belonging to division sixth and last *Schizomycetes.* What may be beyond them, we have now no means of knowing. It seems the border-land of botanical science. But the conviction is borne in upon any who study by aid of optical

*Buffalo Microscopical Club, Dec. 12th, 1882.

instruments, that there is no such thing as empty space, and that cause and effect are not infinitely removed from one another, even though causes may for a long time elude many and careful searches for them, and physicians are still obliged, many times, to confess their ignorance with regard to the causation of disease.

The pearly or gray granulation of miliary tuberculosis, was first described by Mangetus in 1700. A century passed. The year 1800 came and went, and no recorded advance had been made in the knowledge of this small, but almost certain signal of disease and death. The nineteenth century, however, was to be one marked by progress in this, as well as many other directions. In 1809, Bayle described tubercular phthisis. Laennec, in 1819, ten years later, wrote more fully and clearly concerning the morbid phenomena, but overlooked the inflammatory condition. Louis confirmed his observations in these remarkable words: "Nos observations confirment celles de M. Laennec, et que, pour nous, comme pour lui, l'existence des tubercules dans les poumons est la cause, et constitue le caractère propre de la phthisie." This opinion was formed by Laennec and endorsed by Louis without the aid of the microscope. Rokitansky also accepted Laennec's views, and added an observation, the occurrence of multinucleated corpuscles. Thirty years later, in 1849, Loebert, bringing the microscope to bear upon the subject, gave his testimony in favor of the observations and opinions of Laennec and Louis. Not until 1860, did Rheinhardt demonstrate the fact, that tubercle, undergoing cheesy transformation, means inflammation. Virchow endorsed Rheinhardt's discovery, and declared that tubercle might originate from inspissated pus, or *parasites.* Niemeyer now undertook to connect phthisis with pneumonia, but failed. Waldenberg, in 1867, announced that he had succeeded in producing tuberculosis, by inoculation with aniline dyes, boiled tissues and other substances, inert as poisons; apparently confirming the same, by numerous experiments. Villemin, the next year, 1868, inoculated animals with

23

the caseous matter of human phthisis, and produced well marked tuberculosis of internal organs. Discussions, which now arose, led to a more careful histological study of tubercle. A constant pathological element was wanting. Adenoid tissue, giant cells, epithelioid corpuscles, and central caseation were each sometimes present, sometimes absent. Fox and Burdon-Sanderson attributed the disease to the presence of a mechanical irritant. Gerlach, of Berlin, in 1870, as the result of many experiments, declared that non-tubercular matter will not produce tubercle. Orth, in 1873, produced the disease, by feeding tuberculous matter to rabbits. Schneppel was convinced by this of the specific nature of tubercle, and openly advocated it. Buhl, of Munich, in 1874, scarcely eight years before the discovery of Koch's bacillus, showed that tubercles might take their rise in the body, from centres of infection in it, cheesy foci. Schottelius, in 1878, insisted that phthisical granulations may be mistaken for minute capillary pneumonias, or occluded bronchioles. In 1879, Tappenier found that subjecting dogs to the forced inhalation of an atmosphere laden with phthisical sputum, in a state of minute division, was productive of miliary tuberculosis in them, in every instance. The inhalation was continued from twenty-five to fifty-one days. Schottelius varied the experiment by using finely powdered Limburger cheese, powdered brain, bronchitic sputum, &c., with the same result. Wargunin, in April last, published a record of experiments similar to those of Schottelius, and with similar results. Weichselbaum reports, in April or May of this year, that, as a result of eleven experiments upon dogs, infection followed in them after a single inhalation of tuberculous sputum. Baumgarten, in the further support of the assertion of the specific nature of tubercle, inoculated the anterior chamber of the eye with a few drops of blood from a tubercular animal, and a crop of miliary tuburcles was produced in a few weeks. Long ago confusion in the creeds had come. What the ultimate effect of these later discoveries will be in controlling or overcoming the disease, can scarcely, yet, with safety be predicted.

In 1875, the Buffalo General Hospital contained a number of cases of well marked phthisis, as it generally does. The histories were diverse. One had a supposed syphilitic cause. Another, a Swiss, was said to have become a victim to the disease, from change of climate. Another young man had been employed about the railroad station, lifting heavy weights, and had been exposed to sudden changes of temperature, getting heated about his work and cooling suddenly. For another case no cause could be assigned. The patient gained in weight very decidedly, upon cod liver oil and a nutritious diet, but died from phthisis, a few months later. The study of these, and many other cases, with their family histories, led me long ago to doubt the *necessary* heredity of phthisis, except in children born during the time of active development of the disease in the mother. Yet there seems to be, what may properly be called, family predisposition to it. There was also very little in these studies to show the *true cause*, or to suggest a successful treatment of tuberculosis. The developments of the last year, however, throw much light upon an otherwise incomprehensible malady. By histological and pathological research, it was several years ago proven, that the site of infection in the lungs may be variable. The infiltration of tubercle may originate in the wall of a pulmonary vessel, a bronchiole, or a larger bronchial branch, an infundibulum, or alveolus, and in other organs and tissues besides the lungs.

On the 24th of March last, Dr. Robert Koch, formerly of Breslau, now of Berlin, read a paper upon the "Etiology of Tubercle," before the Physiological Society of Berlin, which has been the subject of much comment, by scientific bodies, and the medical and scientific press. This paper may be found in full in the *Berliner Klinische Wochenschrift*, 1882, No. XV, and a resume of it, by Prof. John Tyndall, in the Quarterly Epitome of American Practical Medicine and Surgery, collateral to *Braithwaite's Retrospect*, Vol. 3, No. III. Dr. Koch has established the fact of the presence of the parasitic plant, called bacillus tuberculosis, in phthisical diseases, and proved that the parasite will produce the disease in animals when inoculated.

The proof lies in conclusive culture experiments, extending over as many as ninety days; the animals being inoculated with the last, and presumably purified growth of the bacillus. The plant is white in color, and propagates itself by spores, and not by division. Sporadic propagation is slower than that by division. The perpetuation of the disease is facilitated by the tenacity of life, possessed by the spores. They resist heat and cold, moisture and dryness, and many disinfectants. M. de Korab finds helenine destructive to this bacillus, when out of the body; some other substances have also been found to possess this property. Bacillus tuberculosis is in length equal to one-half to one diameter of a red blood corpuscle, or $\frac{1}{6400}$, to $\frac{1}{3200}$ of an inch. Its width is one-fifth as much. Its spores are arranged at regular intervals. It may be, and probably is, possessed of cilia, which can not be easily shown by any instrument now in use. Cilia have been observed, after so long as five hours consecutive watching for them, in other varieties of bacilli.

There is no longer good room for doubt that a parasitic disease exists, bearing the characteristics of that commonly known and recognized as miliary tuberculosis, communicable, inoculable, and therefore not *necessarily* hereditary. All such diseases are contracted by some persons much more easily than others, and by some families, than others, without amounting to hereditary predisposition. It is estimated that tubercular diseases occasions one-seventh of all deaths of the human race, and one-third of all who die in active middle life. There is as yet no known specific for it.

The minimum of temperature at which the tubercle bacillus can develop and multiply, is 86° F., and the maximum, 104°. Unlike the bacillus anthracis, that of splenic fever, which can flourish freely outside the body in the temperate zone, animal warmth is necessary to the propagation of this newly discovered organism. The spores, however, retain their vitality for a long time without regard to temperature. Change to a favorable locality is all that is necessary to their development.

In a vast number of cases, Koch has examined the matter expectorated from the lungs of persons affected by phthisis, and found in it multitudes of bacilli, while in matter expectorated from the lungs of those not so affected, he has never found it. The expectorated matter in the former cases, was highly infective, nor did drying destroy its virulence. Guinea pigs, infected with matter which had been kept dry for two, four, and eight months, respectively, were smitten with tuberculosis, quite as virulent as that produced by fresh expectoration. The temperature at which it had been kept was not mentioned. Inhalations of dust, containing dried tuberculous matter, are as common as they are dangerous. It is no wonder that the proportion of our death rate is so largely from this single disease.

Prof. R. C. Kedzie, in his annual address as President of the American Public Health Association, Indianapolis, Oct. 17th, made the statement that " It has come to be the belief of scientific and thoughtful men, that preventable sickness, unprevented, is a crime against society, and that preventable death, unprevented, is a crime against God."

Public Health Associations, in this and other countries, are laboring often with almost unlimited means at their command to find the very truth of such matters. There is every precaution taken against mistakes and the deceptions of science. Practical questions are to be solved, and it matters little in what way the solution falls, provided it be a correct one. There are no theories to uphold, no old pledges to theories to redeem, but the utmost freedom, thoroughness and exactness of research. It was under such circumstances that bacillus tuberculosis was detected and is being farther studied.

It is curious to note the growth of an idea, or the steps that lead to a discovery. The first observer of any of these minute organisms, of whom we have any knowledge, was Leuwenhoeck, the so-called father of microscopy. This occurred in 1675. He was astonished, but sufficiently confident of what he saw, to give the description of a number of these forms to the world by publication, describing bacteria and vibrios, so that without his being able to name them with these names, they are now recognized as having been observed by him from his description.

We have no other record of work upon them, except that done by Leuwenhoeck, until a hundred years, lacking two, had passed. In 1773, O. J. Müller endeavored to classify them, making them a group of infusoria, under the name *infusoria crassincula*, establishing two genera, *monas* and *vibrio.* More than fifty papers, considered worthy of publication and preservation, had been written before M. Davaine, in 1859, clearly pointed out that the vibrionens are vegetables. Ehrenberg classed them as animals, so also did Dujardin. Robin pointed out resemblances between bacteria, vibrios and leptothrix. M. Davaine, in 1859, clearly demonstrated that the vibrionens are vegetables, dividing the genus vibrio into eleven species, lineola, tremulans, ingula, prolifer, serpens, bacillus, synxanthus and syncyanus, lactic, butyric and tartaric right. The attention of chemists and pathologists was called to them, and to the entire family of Bacteria, by the researches and discoveries of Davaine, Pasteur and Hallier, concerning their nature and importance. Discussions waxed warm and somewhat warlike. M. Hoffman, in 1869, ten years after the publication of Davaine's memoir, demonstrated: "First, that the bacteria are plants, having a very distinct cellular organization; second, that they can only be classified in accordance with their form and size." M. Cohn, who has spent much time with the bacteria, considers them as belonging to the algae, group schizospores. He notices the absence of chlorophyll. Other botanists consider this characteristic decisive and class them as fungi. It is Nageli who gave them the name of Schizomycetes. In 1876 Cohn and Koch both published important papers, one upon the influence of temperature upon bacteria, their origin and the formation of spores in the bacillus of hay infusion, and in that of charbon, the other being researches upon the bacterium of charbon.

From the publication of Davaine's paper in 1859 up to the year 1880, more than nine times as many publications of papers, &c., upon this subject had been issued than had appeared altogether before that time. It has been a period of great research among these obscure organisms and of great discoveries. Many difficulties have been encountered and overcome. The latest discovery, bacillus tuberculosis is not by any means the least. From a sanitary point of view, it is of the highest importance. It is not necessary at present to prove that there is no other form of phthisis pulmonalis than that produced by this parasitic plant. Sputum, if only suspected, should be disinfected, or, if that prove impossible, taken care of so as to prevent harm. Tuberculous people should themselves take care not to communicate their malady to others. Animals do not need any especial hereditary influence other than that of being animals in order to contract it. It is as well to assume that man does not until the contrary is proven. Inhalations of non-specific dust may, or may not, produce fatal lung affections. It is believed that they do. They can certainly produce irritation, and it is upon a raw or irritated surface that the bacillus is able to find a good foothold.

The examination of the sputum of one hundred and twenty consumptive persons, for bacillus of tubercle, was made during the months of May, June, July and August, last, by Dr. Balmer and Prof. Dr. Fraentzel, and the result published in the *Berliner Klinische Wochenschrift*, Nov. 6. They have arrived at the following conclusions:

1. "The prognosis of a case of tuberculosis may be determined with certainty from the number and degree of development of the tubercle bacilli found in the sputum. All cases, with numerous well developed bacilli, give a bad prognosis. It is bettered in the same proportion that the bacilli decrease. In all cases, which are progressing rapidly, the number of bacilli is monstrous.

2. "The number of tubercle bacilli is by no means constant during the course of tuberculosis; it becomes greater as the breaking down process of the lungs goes on and reaches its maximum at the end of life.

3. "The distribution of the bacilli is not the same in all patients; sometimes they are equally distributed, sometimes they are found in groups.

4. "Their degree of development is also very diverse; in many cases they appear small, scanty and not universally spore-bearing. In these cases their number is always limited.

5. "Such bacilli are found in patients in whom the disease is progressing very slowly, or is stationary, so especially in old caverns closed up by still healthy lung tissue.

6. "In all acute cases of tuberculosis in which the more intense symptoms of the disease are present, such as fever, night-sweats, &c., the bacilli were much larger, and the spore formation in the same clear and indisputable.

7. "Especially were all cases where many bacilli were found accompanied by fever (infection fever). If the fever was lacking, the bacilli were very few and poorly developed.

8. "Very noticeable was the difference between the quantity of bacilli in the sputum from fresh cavities, and that present in the cavern walls themselves. If enormous quantities were found in the former, they would appear in the latter only sparingly.

9. "The sputum appears, therefore, to be a more favorable place of nourishment for the bacilli, than the still living lung tissue.

10. "It can not be asserted that the ingress of oxygen to the cavities of the lungs is the cause of the development of the bacilli, for we found them in just as great numbers in the pus exudation from the closed knee-joint, in the tuberculous inflammation of the joint.

"The evidence of bacilli was present, not only in sputum and in the walls of lung cavities, but also in the tissue, and in the abscess secretion, in tubercular abscess of the lungs, in the wall of intestinal abscess, and in the pus of tuberculous knee-joint inflammation.

"The discovery of bacillus tuberculosis is not alone of use in differential diagnosis of lung diseases, but also in abscesses, inflammation of the joints, &c., is of importance.

"In conclusion, it should be explained, in order to show the difficulty of disinfection of tuberculous sputa, that in sputa almost entirely dissolved by strong potash solution, the coloring of the bacilli is still very clearly present.

"We made the same test also, in the edge layers of sputa, which had lain twenty-four hours in a sublimate solution 1:1,000."

Balmer and Fraenzel used a modification of Ehrlich's modification of Koch's method of examination of sputum lung tissue, &c., for bacillus. Koch's method of examination is as follows:

The tuberculous substance was either spread out upon a cover glass, dried and exposed to heat, or a piece of a tuberculous organ was placed in alcohol, and afterward cut into fine pieces. A particular solution of methylene blue was made, a weak solution of potash being added. The cover glass, covered with tuberculous matter, (or a section of the organ) was then placed in the coloring solution for twenty to twenty-four hours. 200 ccm. of distilled water, were mixed with 1 ccm. of a concentrated alcoholic solution of methylene blue, shaken, and again shaken, after addition 0. 2. ccm. of a 10 per cent. caustic potash solution. One half to one hour sufficed, if the solution were warmed in a water bath up to 72° F.

The cover glass, which comes out a deep blue, is then treated with a concentrated and filtered watery solution of vesuvin for one or two minutes, and is afterward washed with distilled water. The blue of the methylene has visibly changed to brown; under the microscope all the amorphous detritus and fragments of tissue, spread out on the glass, are brown, but the tubercle bacteria remain blue. Bacillus tuberculosis, and bacillus leprosus, are the only bacteria which possess the power of resisting the staining of vesuvin. Bacillus leprosus will take on a certain other coloring, which does not affect bacillus tuberculosis.

Ehrlich's method differs. By means of a dissecting needle, a particle of expectorated matter, about the size of a pin's head, is taken up and spread between two cover glasses, in two exceedingly thin layers. The cover glasses are then separated by sliding one on the other, and left to dry, protected from the dust. After a few minutes, they are dry, and the fixing of the albuminoids and mucin, is proceeded with. For this, they can be warmed for an hour, at 100° or 120° C. or, which is simpler, rapidly passed four or five times through the flame of a spirit lamp. To color the preparations, a saturated solution of aniline is to be made in distilled water, by shaking with the water the excess of aniline, which floats on it, and carefully filtering the whole. To the transparent liquid thus obtained, add, drop by drop, a saturated alcoholic solution of fuchsine, or methyl violet, until a slight opalescence is produced. The preparation should not be immersed in the bath, but be placed in such a manner as to float on it, and to have the surface which is covered with the tuberculous matter in contact with the liquid. After a quarter, to a half an hour, the staining is complete. If examined in this condition, it is seen that the preparation is of such an intense color, that it is impossible to distinguish its elements, and it is decolorized by means of a strong acid. Colorless salts of aniline are thus formed, which are very soluble in water, and disappear by washing with distilled water. The bacteria, not being penetrated by the acids, preserve their color. In order to secure that they alone shall be colored, therefore, it is only necessary to immerse the cover glasses in nitric acid, diluted with twice its volume of water. Nitrous vapors are at once disengaged, and the preparation becomes colorless in a few seconds. The bacilli are red or violet.

Dr. F. Van Ermengen, referring to Koch's method, and Ehrlich's modification of it, describes some additions of his own, which he claims make it absolutely sure in its results. Instead of making a solution of the aniline in water, which only takes up one part in thirty, an alcoholic solution is made, four grammes of liquid aniline, in twenty grammes of alcohol, at 40°, adding an equal quantity of distilled water, and filtering before use. The most stable coloring agents, the author finds to be sulphate of rosaniline and methyl violet B B B B B. The preparations after having been decolorized by dilute nitric acid, are well washed in distilled water. Baumgarten recommends still another method, as simple, and more expeditious than any other. After having spread the tuberculous matter on the cover glass, it is placed in a watch glass and covered with distilled water, to which is added some drops of a 33 per cent. solution of caustic potash. Without any farther preparation, the bacteria may then be recognized under a power of 400–500 diameters, particularly if a light pressure be applied to the cover glasses so as to disengage the bacteria more completely from the detritus which surrounds them. To distinguish them more clearly from the other bacteria, the cover glass may be dried by passing it rapidly two or three times through a flame, and then staining by a concentrated aqueous solution of aniline violet, or other color. The bacteria of tuberculosis are absolutely colorless, while the other bacteria, micrococci, &c., are plainly colored. The whole process only takes ten minutes.

The method practiced by Dr. Osler, of Montreal, is, after drying on the cover glass in the usual manner, to treat with caustic potash, then flow the cover with a solution of aniline violet.

Drs. Balmer and Fraenzel, in the examination of 120 cases, before mentioned, used Kôch's and Ehrlich's methods, with modifications.

They say, "We take a quantity of the sputum, the size of a hemp seed, out of the thick, slimy, purulent mass, with a pair of clean pincers, and without any special selection, place it between two cover glasses, from 0.10 to 0.12 m m thickness, press the cover glasses strongly together, and dry the little sticky particles of sputum, passing one after the other slowly three times through the flame of a Bunsen burner. These cover glasses, for coloring the bacilli, are now laid, swimming upon the coloring solution, and left in the same about twenty-four hours.

"As coloring material it is necessary that we take either gentian violet or fuchsin. From these colors will a solution of 1.0 grm. in 50 grm. aniline water, if possible, be freshly prepared and filtered before using. By aniline water we understand distilled water, to which aniline in excess is added, well shaken together and then the whole carefully filtered. This filtrate we call aniline water. After the cover glasses have lain in the coloring solution as nearly as possible twenty-four hours, the now colored preparations will be taken out with a pair of pincers, washed in distilled water and then for one-half to one minute be laid in dilute nitric acid, (one part pure acid to three of distilled water). In this way must the sticky fragments of the sputum become fully discolored. For this the same time is not always necessary; if the preparation happens to be too thick it must remain longer in the acid until it is discolored. When the preparations are taken out of the acid, they are again washed in distilled water and then laid in another color solution to get the ground color. For those which were in the gentian violet solution, blue colored bacilli, a solution of Bismarck brown; for those in the fuchsine solution, red colored, a solution of methylene blue. These as ground colors need fluidity and are simply concentrated, watery solutions of the color filtered before use. The preparations remain in the ground coloring one-half to one minute, are washed in distilled water and dried between sheets of blotting paper. If they are not soon dry, they can be passed once or twice through the flame of a Bunsen burner. The dry cover glasses will then be laid in a drop of Canada balsam."

Dr. Schmidt, President of the Pathological Society of New Orleans, claims that Koch's bacilli are fatty crystals, because something that he finds and calls by this name dissolves in boiling ether.

At present burning would seem to be the best and only safe method of disposing of sputa of consumptives.

In the *British Medical Journal*, Mr. H. Osborn Bayfield, says that workmen engaged in tinning, where palm oil is used for a flux, inhale the volatilized oil and grow fat. Those previously emaciated or weak rapidly improve, and he suggests a trial of it in phthisis.

The value of certain antiseptic inhalations in most diseases of the respiratory organs has long been established.

The value of a dry pure atmosphere, as a preventive of tuberculosis, was established by Dr. Bowditch nearly twenty years ago. The results of his investigations on the subject were published in the *Atlantic Monthly* as a matter of general public interest. It is precisely the kind of atmosphere in which fungi do not flourish that is least suited to the development of phthisis. In rabbits, the artificial disease has been cured by the inhalation of creosote.

In an early arrest of the disease lies at present the greatest and almost only hope of recovery, although exceptions to this rule are occasionally placed upon record.

Examination of sputa from consumptives now in our Buffalo hospitals by different physicians has revealed the parasite in great numbers. A good one-fifth glass will show their presence, but one-twelfth is more satisfactory.

To a paper in the *Record* of Oct. 28, by Dr. T. E. Satterthwaite, "Bacteria, Magnin," *Journal of the Microscopical Society*, *Braithwaite's Retrospect* and other trustworthy sources I am indebted for many of the facts here collated.

